# Inclusion of Health in Environmental Impact Assessment of Major Transport Infrastructure Projects in Vietnam

**DOI:** 10.15171/ijhpm.2018.36

**Published:** 2018-05-05

**Authors:** Tracy Pham, Emily Riley, Patrick Harris

**Affiliations:** ^1^Faculty of Science, The University of Sydney, Sydney, NSW, Australia.; ^2^Menzies Centre for Health Policy, School of Public Health, Sydney Medical School, The University of Sydney, Sydney, NSW, Australia.

**Keywords:** Health, Transport, Vietnam, Environmental Assessment, Content Analysis

## Abstract

**Background:** Infrastructure spending, especially in the transport sector, is expected to increase rapidly in Vietnam. This boost in transportation investment impacts health. Environmental impact assessments (EIAs) are essential tools for decision-making to reduce and mitigate anticipated impacts of development projects, and integration of health assessment as an essential part of the EIA process has been regulated in many high-income countries. There is, however, limited knowledge about how health is evaluated in these environmental assessments in low- and middle-income countries (LMICs) such as Vietnam.

**Methods:** We did an analysis of EIAs of four major transport projects in Vietnam, applying a six-step coding framework previously used to investigate EIAs in the Australian context.

**Results:** We found that health was inadequately considered in all four EIAs. There was no direct health assessment within the four EIAs due to the lack of formal requirements from either Government or the financing agency, the Asian Development Bank (ADB). Health issues were often identified as risks posed by the projects within the assessment of impacts on environmental conditions. A broader consideration of health was limited. When social outcomes of the projects were present in EIAs, they were often mentioned once without any detailed assessment or linking to health. There was no evidence linking health benefits and shifts towards active travel with the construction of two metro rail projects. Mitigation measures offered in all four EIAs were found to be generic and insubstantial.

**Conclusion:** The health assessments in the EIAs of four transport projects in Vietnam were significantly less detailed than those in Australia, mainly due to the lack of legislative requirements. The lack of health content indicates the need for involvement of health experts in the environmental assessment process, as well as requirements for the health assessment to be integrated in EIA. Our findings suggest there is the need to build capacity both within and outside of government to fully consider the health impacts of infrastructure in EIA practice.

## Background

Infrastructure investments in Vietnam have substantial benefits for the country’s economy and the community. According to a recent report,^1^ the growth rate of infrastructure spending in Vietnam is predicted to be around 9% per year with the investment in the transportation sector accounting for one-third of the total spending. The recent launch of China’s One Belt, One Road (OBOR) Initiative, which proposes to go through many Southeast Asian countries including Cambodia, Laos, Vietnam, Myanmar, Thailand, Malaysia, and Singapore, is expected to have major impacts on the region.^2,3^ It will potentially result in a boost in infrastructure development with a growing focus on transport. However, these developments are accompanied by a range of ensuing impacts on environmental and human health. Transportation affects health by various means such as air pollution, noise and annoyance, traffic accidents and injuries, physical inactivity, disruption in social interaction, and equity.^4-10^

Environmental impact assessment (EIA) has been long recognised as a useful tool to identify, reduce and mitigate anticipated impacts of development projects during their planning.^11,12^ EIA is a planning and decision-making tool for development projects that aims to ensure social and environmental protection. Its power lies in that it is regulated in most countries, including Vietnam.^13^ Internationally, EIA is an important vehicle for considering the impacts of development on human health.^14,15^ However, the lack of comprehensive consideration of health within EIA remains one of the shortfalls of the process.^16-19^ Broadly, health impact assessment (HIA) has been used to identify the health impacts of policy decisions. HIA, albeit its importance, is often voluntary, and separate to EIA.^20,21^ In this study, we looked at the consideration of health within regulated EIA process rather than HIA. Recently published research investigating the coverage of health and health-related issues in the EIA process of four transport projects in Australia showed that health issues were inadequately considered in the EIAs, and mostly in relation to changes to the physical environment in terms of air quality.^22^ This research showed that a broader consideration of health issues, for example, those associated with socio-economic conditions, or the ‘social determinants of health’ such as lifestyle, social influences, availability, access to services and better economic conditions, and health benefits of green spaces are, however, less detailed.^23-25^

The inclusion of health in EIA has been regulated and widely studied in high-income countries (HICs), however, there is inadequate research in this area in low- and middle-income countries (LMICs).^26,27^ The difficulty in accessing EIAs in LMICs is one of the main obstacles due to the lack of transparency of information, and language barriers. Given that the lead author is Vietnamese and understands the local context in Vietnam, we chose Vietnam as a LMIC to replicate the Australian study mentioned previously and apply the framework used to investigate the coverage of health issues in EIAs of major transport infrastructure projects in Vietnam. The findings will be used to test the replicability of the framework on LMICs as well as its usefulness in a global context.

## Methods

This research is a part of a broader research program that uses established social science methods to understand how health and issues pertaining to health are considered in urban planning policy and practice.^27^ Using four transport projects in Vietnam as case studies, we reapplied the analytical framework used to interrogate the content of transport EIAs in the Australian context (Supplementary file 1).^22,27^ We also compared the findings for the Vietnam context against the Australian findings to test the applicability of the framework on LMICs.

### Multiple Case Design and Inclusion Criteria

Multiple explanatory cases studies following methods outlined by Yin were used.^28^ Multiple explanatory cases studies focus on how and why phenomena occur, where each case shows findings which are then either replicated, or not, in other cases. Following the methods, this study developed four cases for in-depth qualitative analyses, and focused on how and why health was included or not in the environmental assessments of four major transport projects. Since the purpose of this study was to replicate the published framework and apply to a Vietnamese context, cases purposively chosen for this study have contextual similarities and differences with four Australian cases. Further, Vietnam’s economy, while experiencing rapid growth, is still at the early stages and much of the capital investment in the country is still derived from foreign sources such as grants or loans, especially for major infrastructure projects. Therefore, different from most HICs there are often two sets of EIA prepared for each transportation project in Vietnam, one to meet the Government’s requirements and the other to fulfill the EIA requirements from financing agencies, such as the Asian Development Bank (ADB), and the World Bank. Therefore, we included cases using the following inclusion criteria:

- The project being assessed has regional transport planning implications and is a Government project.
- The project’s planning is at the state where EIA has been produced and is publicly available.
- The project is likely to have health and well-being impacts on the local community and/or regionally.
- The project receives financial support from ADB given the limited time and resources available for this study.

### Case Sampling

Brief descriptions of the projects being assessed in this research are provided in Table 1 (Supplementary file 2).

**Table 1:** Parameters of the Four Transportation Infrastructure Projects That Were the Focus of This Research

	**Hanoi Metro Rail System Project (Line 3)**	**Ben Luc–Long Thanh Expressway**	**HCMC Metro Rail System Project (Line 2)**	**Ha Noi–Lang Son Expressway**
Project type	Metro rail	Road	Metro rail	Road
Project description	12.5 km-long double-track railway connecting central Hanoi, including an 4 km-long underground section	57.1km-long expressway	11.3 km-long railway includes 9.5 km underground and 2.53 km elevated sections	158.4 km-long expressway
Ownership	People’s Committee of Ha Noi	VN Expressway Corporation	People’s Committee of Ho Chi Minh City	VN Expressway Corporation
Cost (US$)	Initial cost: 991 million; Current cost: 1.4 billion	1.6 billion	Initial cost: 1.37 billion; Current cost: 2.32 billion	1.07 billion
Funded by	Vietnam Government and development loans from ADB, AFD, DGTPE (France), EIB	Vietnam Government (20.9%) and financing as development loan from ADB (39.6%) and JICA (39.5%)	Vietnam Government and development loans from ADB, EIB, KfW	Vietnam Government and loans from ADB
Construction date	2010^ [1] ^	2014	2016^ [2] ^	2016
Expected completion date	2021	2018	2023	2019

Abbreviations: HCMC, Ho Chi Minh City; ADB, Asian Bank Development; AFD, Agence Francaise de Development; DGTPE, Direction Generale du Tresor; JICA, Japan International Cooperation Agency; EIB, European Investment Bank.

In Vietnam, the EIAs prepared for Government appraisal are not available on the Government website. We contacted the Government office to request for the relevant EIAs, however, we did not receive any response. Among the four projects, HN Metro Rail and Ho Chi Minh City (HCMC) Metro Rail required funding from European Investment Bank (EIB), which requires EIAs to be available on the funding agency website. The EIAs of the two projects available on EIB website were prepared based on relevant technical guidelines of EIA from ADB. In the case of Ben Luc–Long Thanh Expressway project, the EIA available on the JICA website was in Vietnamese and it stated in the document that an English version of the document was prepared in compliance with ADB’s requirements ^[3] ^. Therefore, we chose the EIAs prepared for ADB for analysis in this study.

The EIAs as well as ‘environmental assessment guidelines’ developed by ADB were obtained from the ADB website (https://www.adb.org/projects/country/vie and https://www.adb.org/documents/adb-environmental-assessment-guidelines).

### Data Analysis

We applied the framework developed in the Australian study (Supplementary file 1) and reported in detail elsewhere.^22^ The six-step coding framework covers the following categories: case attributes, explicit use of the term ‘health,’ whether a broader understanding of health was shown, the assessment of equity, and comparison against ‘best practice’ criteria. The location and project type of each case were coded under case attributes in the first step. Second, explicit use of the word ‘health’ was coded for each document under a ‘health explicit’ node. The way the term was used in these instances was further interrogated and coded under child nodes, for example ‘health effects,’ ‘health services and facilities,’ and ‘occupational health and safety.’ The term ‘well-being’ was also coded. ‘Additional detail,’ which included the objectives of the projects, sub-issues, mitigation strategies offered, and ‘equity’ as a strategy or an outcome was coded in the third step. Methods used for assessments in the EIAs was coded under ‘methods used’ in the fourth step. Examples of these methods were modelling, risk assessment, and cost benefit analysis. Mitigation measures proposed for issues identified in the EIAs were then coded in step five. Comparisons against ‘best practice’ criteria (ie, provision of community health baseline information, discussion of causal pathways) were coded in the sixth step.

## Results

### “Health” Explicit in Environmental Impact Assessments

The analytic framework applied in this study comprised six steps, and in the second step ‘health’ and ‘well-being’ were coded to analyse how many times they were explicitly mentioned within the EIAs. This can be seen in Table 2.

**Table 2:** Number of Times Terms “Health” and “Well-Being” Appeared in the EIAs of All Cases

	**HN Metro Rail**	**BL–LT Expressway**	**HCMC Metro Rail**	**HN–LS Expressway**
‘Health’ explicit	102	73	85	49
Well-being	2	0	1	3

Abbreviations: HCMC, Ho Chi Minh City; ADB, Asian Bank Development; EIAs, Environmental impact assessments

Despite the size of the EIAs of the four cases in Vietnam being considerably small when compared to similar projects in Australia (200-400 pages long in comparison to over a thousand), the word ‘health’ was explicitly mentioned in each case. In the case of HN-LS expressway, the term ‘health’ was used fewer than in the three other cases. The term ‘well-being’ was rarely used throughout all four documents. In HN Metro Rail EIA, ‘well-being’ was used in a heading in conjunction with health as ‘health and well-being.’

### Consideration of Health Issues in Environmental Impact Assessments

Table 3 below provides the summary of the documentary analysis across the four cases. Within the table, the term ‘insufficient’ has been used to describe when certain elements in terms of technical details or methodology were present or mentioned, but were scant in detail compared to best practice.^22^ The table demonstrates that the way health was framed and addressed was similar across the four cases despite differences in the mode of transport and geographical context.

**Table 3:** Comparisons of EIAs From Documentary Analysis

	**HN Metro Rail System Project (Line 3)**	**Ben Luc–Long Thanh (BL-LT) Expressway**	**HCMC Metro Rail System Project (Line 2)**	**Ha Noi–Lang Son (HN-LS) Expressway**
Evidence of a broad understanding of health	No	Insufficient	Insufficient	Insufficient
Community health baseline/profile (include the existing distribution of mortality, morbidity and health status of affected communities and vulnerable/sensitive sub-groups)	Insufficient	Insufficient. Only information of health facilities was provided	Insufficient	Insufficient
Discussion of the potential associations and causal pathways associated with the project itself	Insufficient	Insufficient	Insufficient	No
Health data and evidence	Insufficient	Insufficient	No	No
Health equity: discussion of the distribution of health impacts across vulnerable/sensitive groups	Insufficient	Insufficient	Insufficient	Insufficient

‏ Abbreviations: HCMC, Ho Chi Minh City; ADB, Asian Bank Development; EIAs, Environmental impact assessments

### Cross-Case Findings

#### Coverage of Health

Across the cases, despite the absence of an explicit health assessment, a mix of health issues was considered. In ADB’s Safeguard Policy Statement (SPS), consideration under ‘Occupational Health and Safety’ and ‘Community Health and Safety’ is required. In ADB’s EA Guidelines, environmental impacts are defined as ‘any change that activities associated with a project may cause in the environment including the impact of any such change on health and socio-economic conditions….’^29^ Yet neither the guide nor SPS require detailed assessment of health. When health was considered in the EIAs, this was limited to direct impacts from changes to the physical environment (eg, discharge of wastewater, air quality, noise and odour; or occupational health hazards). Detailed and comprehensive assessment of mental health impacts of both construction and operation phases of the projects were not included in any of EIAs.

The Metro rail projects were examples of projects where human health could have been included within EIAs as benefits in terms of ‘active walking’ or ‘social cohesion.’ However, the health benefits mentioned in the two EIAs concerned traffic safety, as reductions in traffic accidents resulting from removing private vehicles from roads; and health benefits from improved air quality (and implicitly as ‘community livability’). Equity was considered under the term ‘sensitive receivers.’ This was solely referred to in relation to areas located in the vicinity of construction sites of respective projects rather than populations impacted positively or negatively by the projects.

#### Data and Modelling in the Environmental Impact Assessments

Health was not directly assessed within any of the EIAs, but rather was mentioned as risks posed by the project within the assessment on air quality, air temperature, and noise pollution. In all cases, there was insufficient use of health baseline information, and the information provided in EIAs was limited to the number of health facilities present in the affected regions. These data were presented as part of socio-economic conditions.

#### Mitigation Measures

Mitigation measures offered for health and related impacts were found to be generic and insubstantial, focusing on appointing personnel to ensure the proper implementation of required environmental mitigation measures as stated in EIAs, such as installment of noise barriers, speed control or restriction of fuel use. For instance, in the BL-LT Expressway, one of the mitigation measures offered was ‘undertake monitoring to ensure vehicles moving on this expressway have licenses on ‘compliance to the Vietnam Standard for Vehicle Air Emissions.’

A process called ‘grievance redress mechanism’ (often abbreviated as GRM) was offered as a potential pathway for people adversely affected by a development project to raise their dissatisfactions about the actual and/or perceived impacts during project planning, project implementation and after.^30^

### Case by Case Findings

#### Ha Noi Metro Rail Project

In the HN Metro Rail project EIA, of 102 times the term ‘health’ was mentioned, 72 were in relation to occupational health and safety. Despite the large number of times the term ‘health’ was explicitly mentioned throughout the document, broader consideration of the term was very limited in the EIA. Health impacts of the project identified in the EIA were in regards to changes in physical environment and disruption in family businesses. Health considerations were often described generically as ‘there is concern about the risk to human health.*’*

#### Ben Luc–Long Thanh Expressway

‘Health’ was referred to 73 times throughout the BL–LT Expressway EIA. ‘Health’ was used frequently in relation to ‘public health’ (18 times) and ‘health care’ (16 times). A number of ‘social outcomes’ were considered but without reference to health. For example, conflicts caused by a large influx of workers, separation of communities due to physical barriers created by the project, and impacts on employment opportunities were identified within the EIA. However, impacts on educational opportunities, social capital and cohesion, and the widening of health inequalities were seldom considered. In regards to equity, the term ‘sensitive receivers/receptors’ was used, and mostly focused on populated areas close to construction sites of the project. The risks of the construction phases to children was acknowledged, though this was very limited. Potential effects of the project imposed on children were mentioned once without detailed assessment. Several ‘causal pathways’ linking aspects of the project to health outcomes were identified in the EIA. For instance, the project is linked with improvements in living and educational conditions in regards to changing social roles of women. However, no supporting evidence was provided for these claims.

#### Ho Chi Minh City Metro Rail

Of the 85 references to ‘health’ in the HCMC Metro Rail EIA, 52 were in relation to health and safety combining ‘health and safety,’ ‘health and safety hazards,’ and ‘occupational health and safety.’ Cumulative health-related impacts identified within the EIA of the HCM metro rail project were described as ‘positive long-term benefits’ (ie, improvement of urban air quality, public health, safety and travel time savings). Even though improved health was not explicitly mentioned within the EIA objectives, health was considered throughout the document. It is stated that one of the long-term benefits of the project was ‘preserving community livability and green space, encouraging pedestrian and bicycle traffic along the corridor and reducing greenhouse gas (GHG) emission.’ However, the health benefits of shifting towards active travel were not detailed. There was evidence linking health benefits and public transportation included in the EIA, mainly towards reducing air pollution, traffic accidents and congestion. Despite this, and similar to the other cases, health impacts were only mentioned in relation to the physical environment, with social factors related to health not considered. One example of a health consideration was the comparison against WHO air quality standards associated with the increase in mortality rate from exposure to degraded air quality.

#### Ha Noi–Lang Son Expressway

‘Health’ was used explicitly 45 times in the HN–LS Expressway EIA. Of these references, 18 were in relation to health facilities. In the case of the HN-LS Expressway project, despite the small number of times the term ‘health’ was explicitly mentioned in comparison with the other cases, equity was considered in slightly greater detail. As the HN–LS Expressway project affected indigenous people, equity was considered in the EIA focusing on the social and psychological impacts on indigenous people. Consideration of health risks posed to women and children was also included. These considerations, however, were limited to one-time statements without detailed assessment. Existing distribution of mortality and morbidity were not, however, available.

Unlike the other three cases, the Department of Health – at both a provincial and national level – was involved in a feasibility study of the project. Their involvement was limited though to providing surveys for information on community health and health facilities, focusing on the status diseases such as HIV/AIDS and sexually transmitted infections in the region. The HN-LS Expressway project also asserted that ‘ill health can also result from the disruption patterns, and hence, traditional sources of nutrition.’ However, the link between a disruption in sources of nutrition and ill health was not expounded.

## Discussion

This study is a replication of a published comprehensive framework to analyse the coverage of health in EIAs in Australia in the Vietnamese context. Our findings demonstrate the utility of this framework for LMICs. Our analysis shows that health was mentioned in all four cases despite health not being officially required as part of the EIA process. However, in relation to the known evidence linking public health and transport policy decisions,^31^ as well as international calls for the inclusion of health in EIA,^27,32^ we find that these considerations are ‘insufficient’ due to several reasons: potential health risks were identified but direct health assessment was absent; social aspects were under-considered in terms of detail; the community health baseline was not available except for information of health facilities, which is insufficient for informing health predictions; and a lack of causal pathways considered changes to the environmental or social conditions and health outcomes.

In the Australian cases from the original study,^22^ in three out of four projects the proponent was required to assess health risks associated with air quality, soil quality, and noise and vibration in the EIAs. The Vietnamese EIAs analysed in this study were much less detailed than those included in the Australian study. The differences between Australia and Vietnam could be explained by many factors, these include the differences in the state of economic development, the scale of infrastructure, the legislative process and management systems.^19^

The absence of a health section or technical working paper in all four EIAs in this study was unsurprising, given health was not directly required in either of the two guidelines for preparation of EIA in Vietnam. The predominant view of human health within the EIAs is that it is mainly understood as the environmental risks to health, as well as ‘Occupational Health and Safety.’ This further demonstrated the importance and influence of regulations on the way health was framed and assessed.

Another major finding was that health consequences of the change in transport mode to a multi-modal transport system with the construction of metro rail lines was not considered in any detail in the EIAs. Multi-modal transport system refers to a system that has various travel modes, such as walking, cycling, motorbike, automobile and public transit, and connection among modes. In a multi-modal transport system, ‘modal split’ of each travel mode was then calculated by the percentage of passengers or of the number of trips using that particular travel type. Given the extensive empirical research linking cycling and walking with benefits to health and well-being,^33-36^ health was not explicitly mentioned within the objectives of the HN Metro Rail project is insufficient. In the two public transport EIAs, the health implications of the project were mainly discussed in terms of improved air quality and reduced traffic congestion. Our analysis suggested that health could have been inserted as a core aspect of these assessments. This, given the above focus, could have been framed about the benefits of the proposal as a whole, particularly in its operation phase. Alternatively, given the current transport conditions in Vietnam, with an extremely high share of motorcycles (over 80% of transport mode being motorcycles) and a low modal split of public transportation,^37-39^ the shift to walking and cycling could potentially result in increased traffic accidents. Vietnam, particularly in HCMC and HN, in recent years has observed government’s effort to shift transportation behavior from private vehicles to public transport with the development of bus and metro rail systems. In the last year, for instance, the HN Peoples Committee announced a plan to ban motorcycles in downtown HN in 2030.^40^ This suggests that the inclusion of health impacts in transport policy and planning is crucial for Vietnam.

### Implications

The framework used in this study could be used by practitioners to assess the quality of health’s inclusion in EIA system for major development projects in Vietnam in general. Through this, additional health input can be used for the production of technical guidelines on the assessment of health. Several recent activities in Vietnam illustrates the recognition of the importance of considering the health impacts of development projects. A National Environmental Health Action Plan (NEAP) was prepared as collaborative work between the Ministry of Natural Resources and Environment (MONRE) and the Ministry of Health (MoH). In April 2017, the second ASEAN conference on HIA with the theme ‘Health Impact Assessment for Public Health Policy’ took place in Vietnam. Additionally, our replication of the documentary analysis framework in the Vietnamese context suggests the framework may be useful internationally. The applicability of such frameworks is particularly important given recent announcements about infrastructure construction globally and in the Asian context especially, such as China’s One Belt One Road Initiative.^2,3^

In addition, our findings can serve as a catalyst for integrating health assessment within EIA to improve the effectiveness of EIA process in the quest for sustainability. Current approaches towards sustainability in Vietnam mostly focus on environmental protection and economic growth.^41^ However, the problem of sustainability does not only lie in an effort to strike balance between economic growth and environmental protection, but also requires the thorough consideration of anticipated impacts of development projects on population health. The importance of ensuring health and well-being is one of the sustainable development goals set out by United Nations.^42^ Recently there has been interest from multilateral lending institutions, ADB included, towards balancing health with environmental and social issues on the sustainability triangle.^43^ Our findings could be utilised for further research towards a more practical pathway - a comprehensive integration of health assessments in EIA in LMICs, particularly in Vietnam.^44^

### Limitations

There are some limitations to the analysis. First, there was no formal requirement for health to be included in the EIA process; therefore, the analysis of the EIAs against the best practice categories for including health in EIA was limited. Second, with health assessment not being required in EIA process, there was difficulty in approaching professional stakeholders to speak about the health inclusion. Within the short timeframe for the research, we were only able to conduct one interview, this important information from stakeholders was, therefore, excluded from our results. In addition, due to the availability of publicly accessible EIAs, the small size of sampling in this study may not fully capture how health is situated in EIAs in Vietnam. EIA has only been legislated in Vietnam since 2011 under the Decree 29/2011/ND-CP^ [4] ^. EIAs chosen to be accessed in this study is considerably large and detailed in comparison to existing available EIAs. As a result, we can conclude that the extent of consideration of health issues found in four EIAs is close to current practice in the country.

## Conclusion

Given the Vietnamese Government’s commitment towards Sustainable Development Goals and improving public health and transport infrastructure, Vietnam is an important LMIC case to understand the quality of EIA as a policy mechanism for assessing the health impact of infrastructure projects. We have shown that health issues were not comprehensively considered in the EIA process. Our findings suggest limited awareness and appreciation of the health-related implications of infrastructure development within the EIA process in Vietnam. However, the inclusion of health in EIA is a relatively novel development. There was recognition of a broad consideration of health in EIA practice in the cases but crucially the assessment of health was not developed in detail. Our findings suggest there is a need to build capacity both within and outside of government to fully consider the health impacts of infrastructure in EIA practice. Given the significant role of international communities for strengthening the EIA system and its implementation in Vietnam,^45,46^ we recommend that the financing agencies and international bodies, such as ADB, World Bank, and WHO develop a comprehensive framework particularly for LMIC settings for assessing impacts of transport infrastructure on health, and formally require the assessment to be included as part of the project approvals process.

## Acknowledgements

This study was conducted as capstone research project for Master of Sustainability, the University of Sydney, under supervision of Emily Riley and Patrick Harris at Menzies Centre for Healthy Policy, Sydney, NSW, Australia. Patrick Harris is funded by the Australian National Health and Medical Research Council, Australia (APP1090644).

## Ethical issues

Ethics approval was granted by the Human Research Ethics Committee, the University of Sydney, Sydney, NSW, Australia. The ethics approval number is 2017/317.

## Competing interests

Authors declare that they have no competing interests.

## Authors’ contributions

TP designed the research with PH. TP conducted the analysis and wrote up this article with ER. ER and PH reviewed this article and provided additional input.

## Authors’ affiliations

^1^Faculty of Science, The University of Sydney, Sydney, NSW, Australia. ^2^Menzies Centre for Health Policy, School of Public Health, Sydney Medical School, The University of Sydney, Sydney, NSW, Australia.

## Endnotes


[1] The construction stage of the HN Metro Rail System Project first started in 2006 and expected completion was dated in 2010. However, due to unexpected complexity in resettlement and lack of funding, the construction was disrupted and restarted in September 2010. An EIA was prepared and submitted to ADB prior to the construction in 2010. An updated version of this EIA was made available on ADB’s website in 2013.

[2] At the time of writing this, HCMC Metro Rail System project is undergoing resettlement phases and construction stage is expected to start in 2018 and to be completed in 5 years.

[3] Translated from: “Do dự án cần vay vốn của Ngân hàng Phát triển Châu Á (ADB) nên ADB yêu cầu thực hiện ĐTM theo quy định trong Chính sách An toàn và Môi trường ban hành tháng 6 năm 2009 (ADB’s Safeguard Policy Statement, 2009). Theo quy định này một báo cáo ĐTM (bản tiếng Anh) đã được đơn vị tư vấn lập, đã được Hội đồng của ADB xem xét thẩm định tại trụ sở ADB ở Manila, Cộng hoà Philippines (6/2010).” Available at: https://libportal.jica.go.jp/library/Data/DocforEnvironment/EIA-EPC/SoutheastAsia/VietNamExpressway/BenLuc-LongThanhEIA.pdf.

[4] Details of the Decree 29/2011/ND-CP is available at: http://moj.gov.vn/vbpq/en/lists/vn%20bn%20php%20lut/view_detail.aspx?itemid=10586.


## Supplementary Files


Supplementary file 1. Coding Framework for investigation the inclusion of Health in Environmental Impact Assessment.^22^

Supplementary file 2. Location of the assessed projects.

## Key messages

**Implications for policy makers**

- Policy-makers should set the requirements to comprehensively include health in the environmental impact assessment (EIA) process for major transport infrastructure projects.
- EIAs in Vietnamese context should take into account the health consequences of the shift to multi-modal transportation.
- The findings support the use of our content analysis framework as an audit tool for assessing the quality of health coverage in EIA in low- and middle- income settings.
- Financing agencies and international bodies (eg, the Asian Development Bank [ADB], the World Bank and World Health Organization) should develop a comprehensive framework for assessing impacts of transport infrastructure on health, particularly for low- and middle-income country (LMIC) settings and require the assessment to be included as part of the project approvals process.

**Implications for the public**

This research illustrates how health was situated in environmental impact assessment (EIA) of four major transport infrastructure projects in Vietnam. Our findings suggest that there is certain recognition of broader understanding of health in EIA practice, however, a more comprehensive assessment of health issues has to be included in order to assess consequences of transportation decisions to public health.
