# Supplementary material for: Inclusion of Health in Environmental Impact Assessment of Major Transport Infrastructure Projects in Vietnam
Source: Int J Health Policy Manag. 2018 May 5;7(9):828–35. doi: 10.15171/ijhpm.2018.36 (PMC6186477; doi:10.15171/ijhpm.2018.36)
Supplement: Supplementary file 1 — Coding Framework for investigation the inclusion of Health in Environmental Impact Assessment.22 [file ijhpm-7-828-s001.pdf]

Supplementary 1. Coding Framework for Investigation the Inclusion of Health in Environmental Impact Assessment[22]

Approach	Coding for What?
Step 1: Attribute coding	Attributes of the case found in the EIA and requirements (eg, expressway, metro rail)
Step 2: 'Health' explicit	'Health' and 'well-being' and derivatives of these which mention either word
Step 3: Additional detail	**Objectives** (for the project being assessed/ for the EIA)<br>**Topics** (in the EIAs *against chapters, headings)*<br>**Sub-issues/ impacts/ outcomes** – eg, air quality, noise, social well-being<br>• Health outcomes (mental health, mortality, etc)<br>• Environmental outcomes (reduced pollution, reduced noise, reduced traffic congestion, etc)<br>• Social outcomes (well-being, isolation, etc)<br>• Economic outcomes (business, employment, etc)<br>• Behavioural outcomes (increased physical activity, reduced behavioural risk factors, etc)<br>**Strategies**<br>• Structural<br>• Individual behavior<br>**Equity** (strategy or outcome)

Step 4: Methods used<br>• in the health assessment<br>• in assessments of other issues	• Primary data collection – qualitative, quantitative, survey<br>• Stakeholder analysis<br>• Baseline<br>• Secondary data (statistics, documents, literature)<br>• Risk assessment – quantitative and qualitative<br>• Cost benefit analysis<br>• Modelling<br>• Other
Step 5: Mitigation measures<br>• in the health assessment<br>• in assessments of other issues	What mitigation measures proposed in EIAs?
Step 6:<br>Best practice approach to technical inclusion of health in EIA<br><br>Including and assessment of the	1. **Community health baseline/profile** (incl the existing distribution of mortality, morbidity and health status of affected communities and vulnerable/ sensitive sub-groups).<br>2. **Causal pathways:** [Evidence-informed?] discussion of the potential associations and causal pathways from a 'project aspect' (project process or activity) leading to a possible change in one or more health determinants that are likely to cause a change in one or more health outcomes (eg, communicable disease, non-communicable disease).

quality of the information used	**3. Health data and evidence:** Use of health impact research evidence, qualitative and quantitative, to identify causal pathways and the significant of a health impact.<br>**4. Health equity:** Discussion of the possible interactions between project aspects, health determinants, health outcomes and health equity. Discussion of the distribution of health impacts across vulnerable/ sensitive groups (eg, lower socio-economic groups, women, children)

Abbreviation: EIA, environmental impact assessment.

Note: The coding framework is reproduced with permission from the authors of the cited article.
