# Supplementary material for: Inclusion of Health in Environmental Impact Assessment of Major Transport Infrastructure Projects in Vietnam
Source: Int J Health Policy Manag. 2018 May 5;7(9):828–35. doi: 10.15171/ijhpm.2018.36 (PMC6186477; doi:10.15171/ijhpm.2018.36)
Supplement: Supplementary file 2 — Location of the assessed projects. [file ijhpm-7-828-s002.pdf]

**Supplementary file 2.** Location of the Assessed Projects

**2A.** HN Metro Rail (line 3)

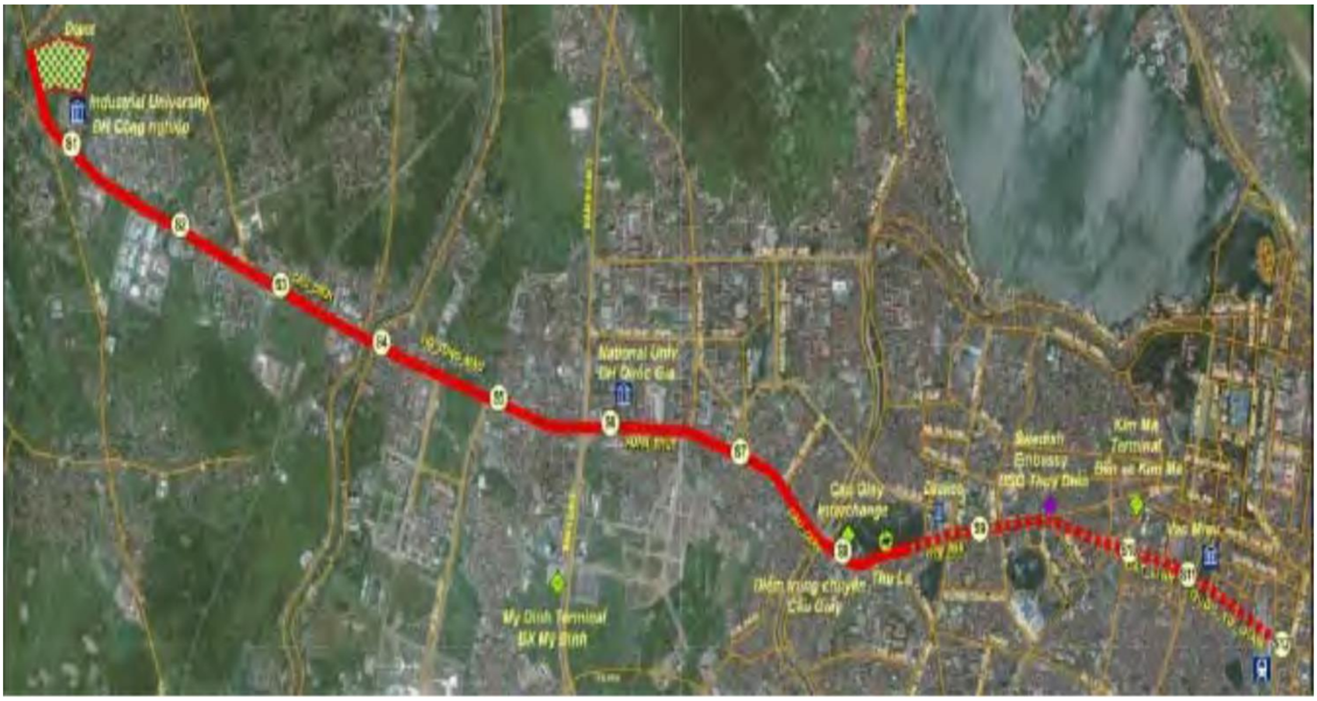


Source: HN Metro Rail (line 3) EIA 2013, ADB.

**2B.** Ben Luc – Long Thanh Expressway

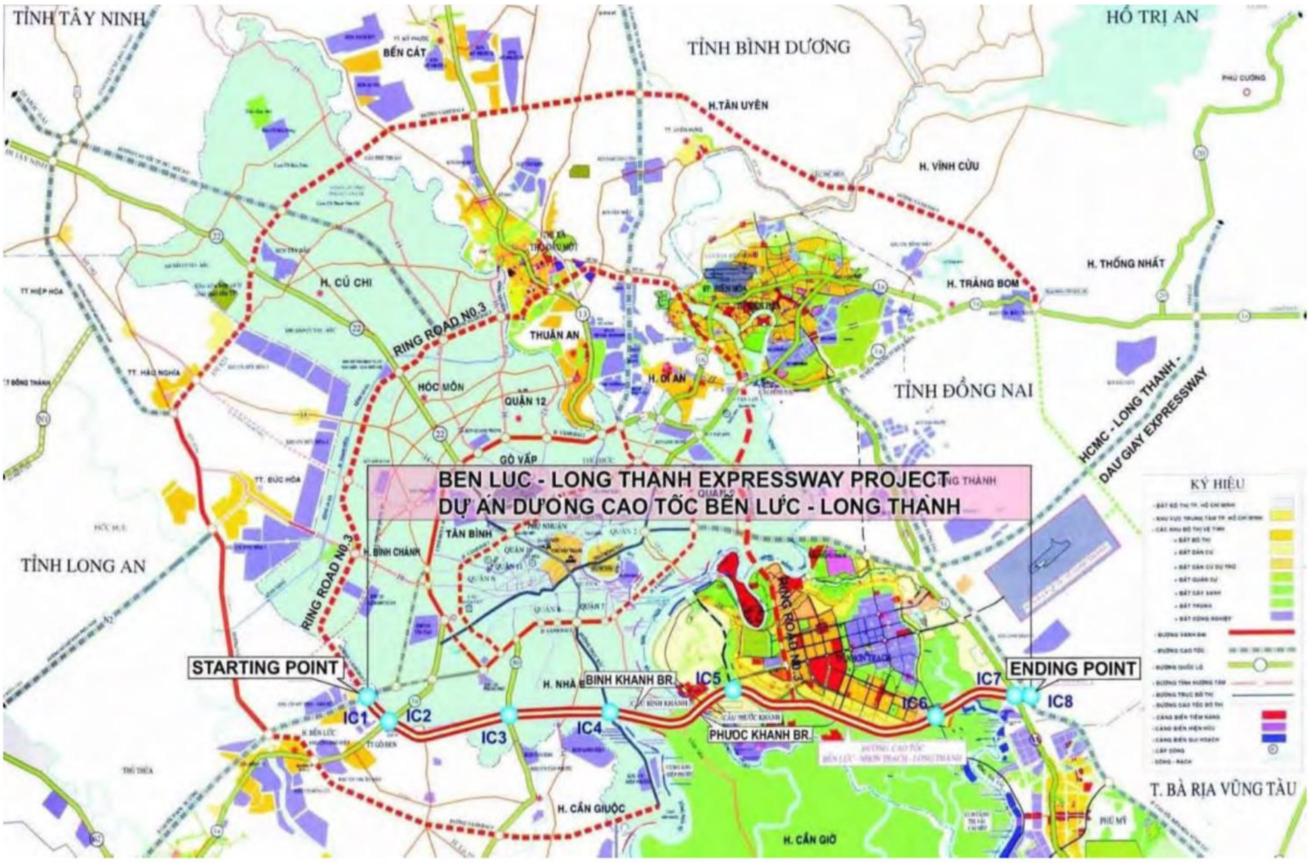


Source: Ben Luc – Long Thanh Expressway EIA 2010, ADB.

**2C.** HCMC Metro Rail (line 2)

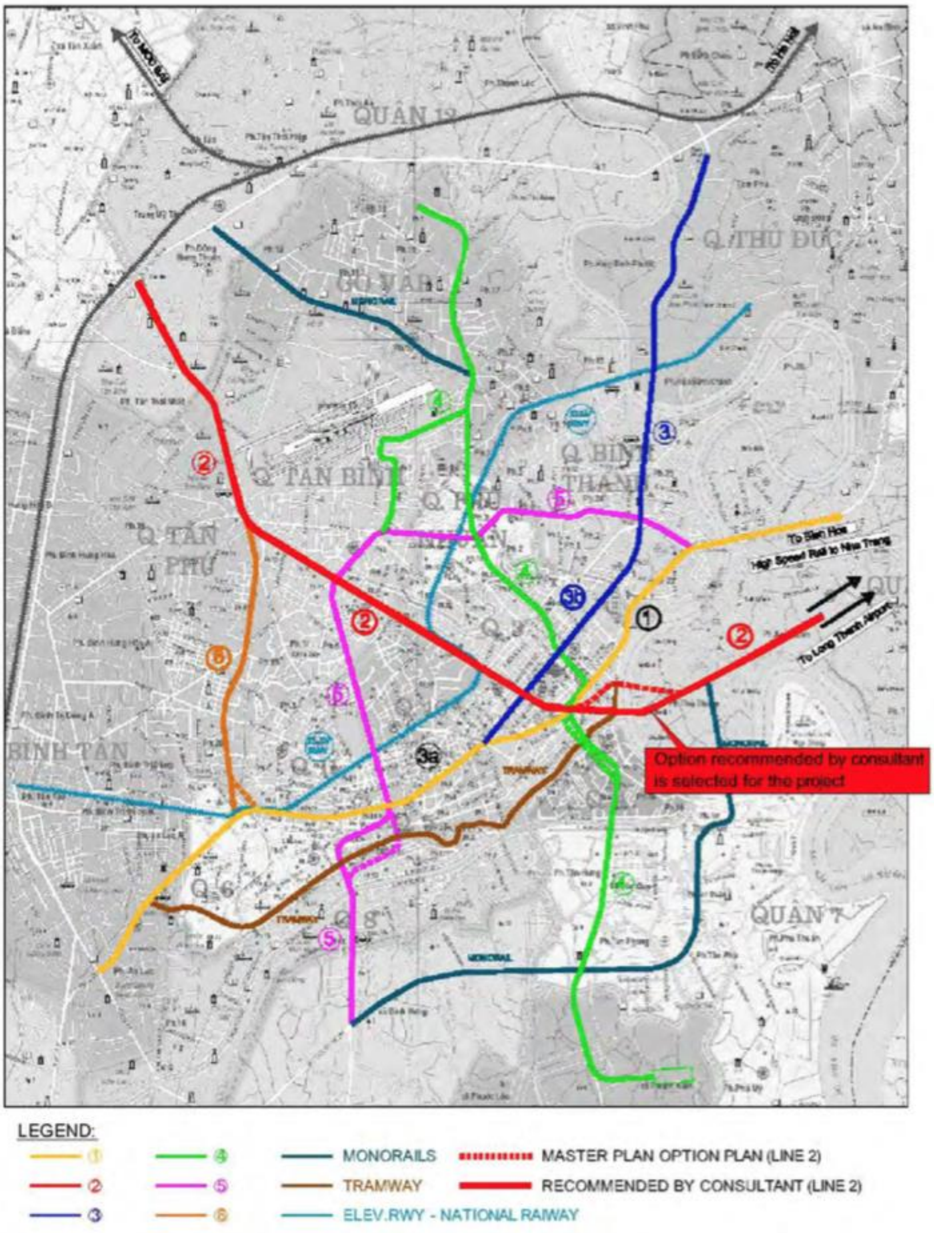


Source: HCMC Metro Rail (line 3) EIA 2012, ADB.

**2D.** Ha Noi – Lang Son Expressway

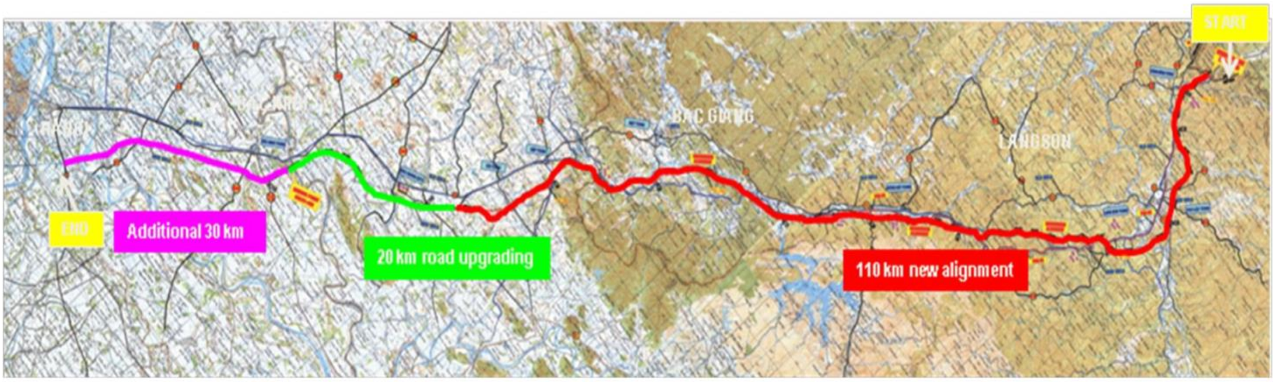


Source: Ha Noi – Lang Son Expressway EIA 2011, ADB.

Abbreviations: ADB, Asian Bank Development; EIA, environmental impact assessment.
